# Comprehensive Analysis of the Expression and Prognosis for GBPs in Head and neck squamous cell carcinoma

**DOI:** 10.1038/s41598-020-63246-7

**Published:** 2020-04-08

**Authors:** Zeng-Hong Wu, Fucheng Cai, Yi Zhong

**Affiliations:** 1 Department of Otorhinolaryngology, Union Hospital, Tongji Medical College, Huazhong University of Science and Technology, Wuhan, Hubei China; 2 Department of Infectious Diseases, Union Hospital, Tongji Medical College, Huazhong University of Science and Technology, Wuhan, 430022 China; 3 Department of Pediatrics, Union Hospital, Tongji Medical College, Huazhong University of Science and Technology, Wuhan, 430022 China

**Keywords:** Diagnostic markers, Risk factors

## Abstract

Guanylate binding proteins (*GBPs*) belongs to the interferons (IFNs) induced guanylate-binding protein family (Guanosine triphosphatases, GTPases) consisting of seven homologous members, termed *GBP1* to *GBP7*. We used multidimensional survey ways to explore *GBPs* expression, regulation, mutations, immune infiltration and functional networks in head and neck squamous cell carcinoma (HNSCC) patient data based on various open databases. The study provides staggered evidence for the significance of *GBPs* in HNSCC and its potential role as a novel biomarker. Our results showed that over expressions of 7 *GBPs* members and multivariate analysis suggested that N-stage, high expressions of *GBP1* and low expression of *GBP6/7* were linked to shorter OS in HNSCC patients. In addition, B cells of immune infiltrates stimulant the prognosis and might have a medical prognostic significance linked to *GBPs* in HNSCC. We assume that *GBPs* play a synergistic role in the viral related HNSCC. Our results show that data mining efficiently reveals information about *GBPs* expression in HNSCC and more importance lays a foundation for further research on the role of *GBPs* in cancers.

## Introduction

Head and Neck Squamous Cell Carcinoma (HNSCC) is a common head and neck malignancy that originates from lips, mouth, paranasal sinuses, oropharynx, larynx, nasopharynx, and other pharyngeal cancers^1^. As the sixth most common type of malignant tumors, with more than 655,000 new cases and 90,000 deaths every year^2^. Currently, smoking, drinking, and human papillomavirus (HPV) infection are considered risk factors for HNSCC occurrence and prognosis^3^. Unfortunately, the 5-year survival rate is still below 50% due to the usual lack of early symptoms when HNSCC is detected early, while the survival rate is reduced to 35% due to local recurrence and metastasis^4^.

Once in advanced stages, treatment can notably effect organ function and harm the structures involved in speaking and swallowing, causing devastating results in patient’s quality of life^5,6^. Studies reported that lots of HNSCC not only has experienced epithelial-to-mesenchymal transition (EMT) but also presents a mesenchymal-like (ML) phenotype and thus effect the drug resistance, tumor migration or tumor growth^7^. The incidence and development of HNSCC is a complex process involving multiple molecules. Guan *et al*. found long non-coding RNA H19 and its mature miR-675 were significantly high level in two HNSCC cell lines as well as a cohort of 65 primary tumor samples^8^. Wu *et al*. reported that *SUZ12* protein was particularly overexpression in primary HNSCC samples and is up-regulated significantly associated with cervical node metastasis, overall survival and disease-free survival^9^. Reed *et al*. believed that inactivation of the p16 tumor suppressor gene is a common event in HNSCC^10^. In spite of advances in combine chemotherapy, radiation, and surgery during the past decades have fundamentally improved survival rate of the HNSCC patients, many patients increase secondary tumors, recurrences or metastasis after treatment and thus becomes a challenging problem and leading to therapeutic failure^11^.

Guanylate binding proteins (GBPs) belongs to the interferons (IFNs) induced guanylate-binding protein family (Guanosine triphosphatases, GTPases) consisting of seven homologous members, termed *GBP1* to *GBP7*^12^. Until now, seven different *GBPs* and six in mouth have been identified and human *GBPs* show a high level of homology. GTPases are involved in process of signal transduction, cell proliferation, differentiation and intracellular protein transportation^13,14^. Inside host cells, *GBPs* quickly assemble into big antimicrobial defense complexes that fight various bacterial pathogens^15^. Studies found the immune roles of human *GBPs* in regulating not only pyroptosis, but also apoptosis^16^. Most previous studies the function of *GBPs* in bacterial pathogens, but more and more studies explore the role of *GBPs* in tumor mechanism. Study found that *GBP1* acts specifically as a tumor suppressor in colorectal carcinoma and the reduction of *GBP1* expression might imply cancer evasion from the IFN-γ-dominated Th1 immune response^17^. Nevertheless, the mechanism by which *GBPs* are depressed or activated still remained unclear in the development and progression of HNSCC. Until now, a comprehensive bioinformatics analysis has not yet to analysis the potential role of *GBPs* in HNSCC. we explored the mutations and expressions of various *GBPs* based on thousands of variations in copy numbers or gene expressions in patients whom with HNSCC in detail to provide new insights into the potential functions, expression patterns, molecular mechanisms and distinct prognostic underlying *GBPs* regulation.

## Results

### Over-expression of different GBPs family members in HNSCC

**Figure 1:**
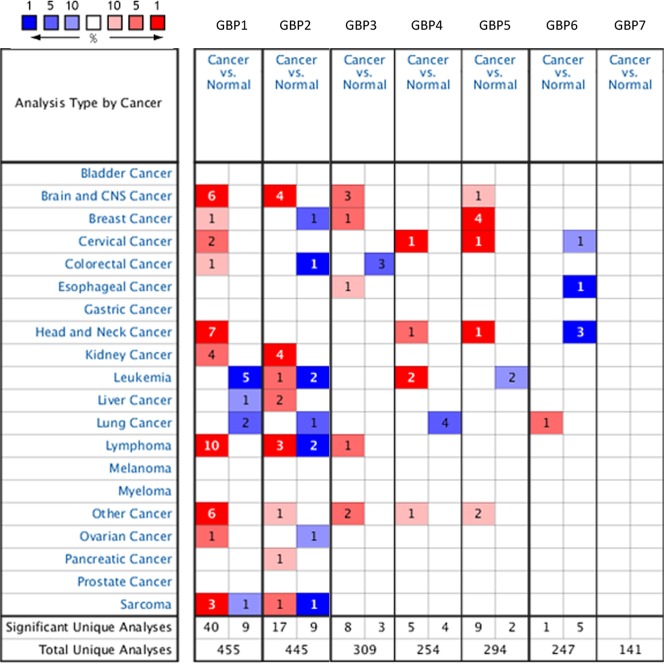
The transcriptional levels of *GBPs* in cancers and normal samples and significantly higher mRNA expressions of *GBP1/4/5* were found in HNSCC tissues in multiple datasets. Redder means higher expression and bluer means lower expression, the analysis which can give us an understanding of the expression of *GBPs* family in different cancer types. (ONCOMINE Database).

**Figure 2:**
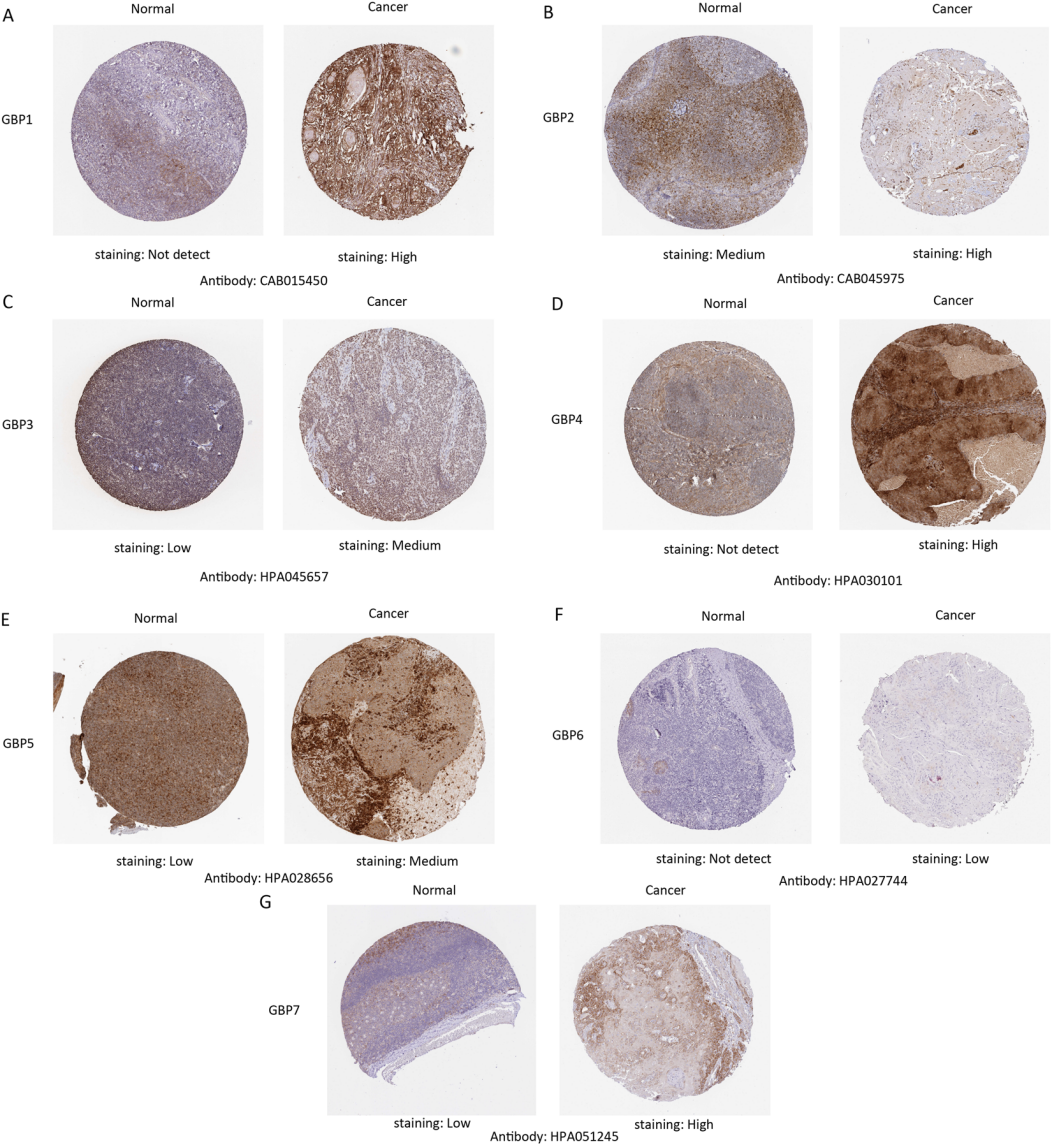
Representative immunohistochemistry images of distinct *GBPs* family members in HNSCC tissues and normal tissues (Human Protein Atlas Database).

In order to explore the potential therapeutic value and distinct prognostic of different *GBPs* members in HNSCC patients, the expression *GBPs* were explored by ONCOMINE database, TCGA database and HPA database. We first contrast the transcriptional levels of *GBPs* in cancers and normal samples and significantly higher mRNA expressions of *GBP1/4/5* were found in HNSCC tissues in multiple datasets (Fig. 1). In Peng Head-Neck statistics^18^, *GBP1* over-expression was found in oral cavity squamous cell carcinoma tissues compared with normal tissues with a fold change of 7.854 (*P* = 4.15E-24), while Talbot^19^ observed 2.962-fold increase in *GBP1* mRNA expression in tongue samples (*P* = 3.10E-12), Ginos^20^ found 5.397- fold increase in *GBP1* mRNA expression in HNSCC tissues (*P* = 2.70E-13), Estilo^21^ found 4.286- fold increase in *GBP1* mRNA expression in tongue tissues (*P* = 3.51E-9) and Ye^22^ found 4.450- fold increase in *GBP1* mRNA expression in tongue tissues (*P* = 5.39E-5, Table 1). Significant up-regulation of *GBP4* was also found in HNSCC tissues compared to normal tissues. In Peng Head-Neck statistics^18^, *GBP4* over-expression was found in oral cavity squamous cell carcinoma tissues compared with normal tissues with a fold change of 3.256 (*P* = 2.06E-12). Similarly, In Peng Head-Neck statistics^18^, *GBP5* over-expression was found in oral cavity squamous cell carcinoma tissues compared with normal tissues with a fold change of 14.065 (*P* = 3.61E-26), while Ye^22^ observed 3.376-fold increase in *GBP5* mRNA expression in tongue samples (*P* = 1.53E-4). We then tried to analysis the protein expression patterns of *GBPs* in HNSCC by the HPA database. As the result shown *GBP1/4/5* proteins were not expressed in normal head and neck tissues, whereas high and medium expressions of them were observed in HNSCC tissues. In addition, low protein expressions of *GBP3/7* were expressed in normal tissues, while medium and high protein expressions of them were observed in tumor tissues. Moreover, medium protein expression of *GBP2* was observed in normal tissues and high expression in HNSCC tissues **(**Fig. 2). However, no expression and low protein expression of *GBP6* was observed in normal and tumor tissues. In total, our results suggested that transcriptional and proteinic expressions of *GBPs* were over-expressed in patients with HNSCC.

**Table 1:** The Significant Changes of *GBPs* Expression in Transcription Level between Different Types of HNSCC and Normal Tissues (Oncomine database).

*GBPs*	Type of Breast Cancer versus Normal Breast Tissue	Fold Change	*P* Value	t Test	Source and/or Reference
*GBP1*	Oral Cavity Squamous Cell Carcinoma	7.854	4.15E-24	16.990	Peng Head-Neck statistics^18^
	Tongue Squamous Cell Carcinoma	2.962	3.10E-12	9.029	Talbot Lung statistics^19^
	Head and Neck Squamous Cell Carcinoma	5.397	2.70E-13	10.813	Ginos Head-Neck statistics^20^
	Tongue Squamous Cell Carcinoma	4.286	3.51E-9	7.071	Estilo Head-Neck statistics^21^
	Tongue Squamous Cell Carcinoma	4.450	5.39E-5	3.433	Ye Head-Neck statistics^22^
*GBP2*	Oral Cavity Squamous Cell Carcinoma	1.636	3.53E-8	6.125	Peng Head-Neck statistics^18^
	Oral Cavity Squamous Cell Carcinoma	1.692	0.035	2.151	Toruner Head-Neck Statistics
*GBP3*	Oral Cavity Squamous Cell Carcinoma	1.792	4.54E-9	6.542	Peng Head-Neck statistics^18^
*GBP4*	Oral Cavity Squamous Cell Carcinoma	3.265	2.06E-12	8.363	Peng Head-Neck statistics^18^
*GBP5*	Oral Cavity Squamous Cell Carcinoma	14.065	3.61E-26	16.300	Peng Head-Neck statistics^18^
	Tongue Squamous Cell Carcinoma	3.376	1.53E-4	3.997	Ye Head-Neck statistics^22^
*GBP6*	NA	NA	NA	NA	NA
*GBP7*	Oral Cavity Squamous Cell Carcinoma	1.636	3.53E-8	6.125	Peng Head-Neck statistics^18^

NA, not available; HNSCC: head and neck squamous cell carcinoma.

### Association of mRNA expression of different GBPs family members with clinicopathological parameters of HNSCC patients

**Figure 3:**
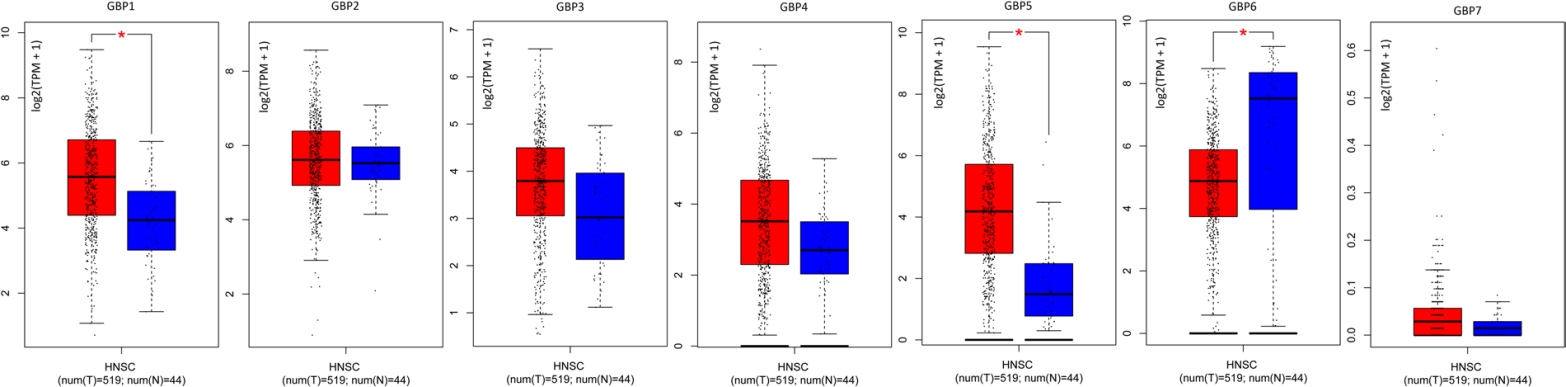
The box plot Expression of *GBPs* in HNSCC. The box color of red indicates tumor and blue indicates normal. The method for differential analysis is one-way ANOVA, using disease state as variable for calculating differential expression and asterisk means statistically significant, with each dot representing a distinct tumor or normal sample. (GEPIA Database; TPM: Transcripts Per Million; T: Tumor; N: Normal).

**Figure 4:**
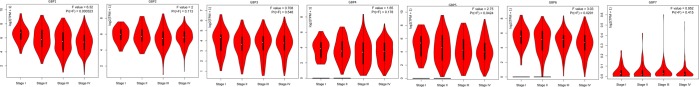
The expression of *GBPs* with cancer stage (Stage I~Stage IV) for HNSCC and we found *GBP1/5/6* groups significantly varied, but *GBP2/3/4/7* groups did not significantly differ. The method for differential gene expression analysis is one-way ANOVA, using pathological stage as variable for calculating differential expression. The larger *F*, the better the study fit and *P*-Value < 0.05 as statistically significant. (GEPIA Database).

Using the GEPIA dataset, we first contrast the mRNA expression difference of *GBPs* between cancer and normal tissues. The results demonstrated that the expression of *GBP1* and *GBP5* were higher in HNSCC tissues, while the expression level of *GBP6* was lower in cancer tissues (Fig. 3**)**. Moreover, we also explored the expression of *GBPs* with cancer stage for HNSCC and we found *GBP1/5/6* groups significantly varied, but *GBP2/3/4/7* groups did not significantly differ (Fig. 4).

### Prognostic value of mRNA expression of GBPs in HNSCC

**Figure 5:**
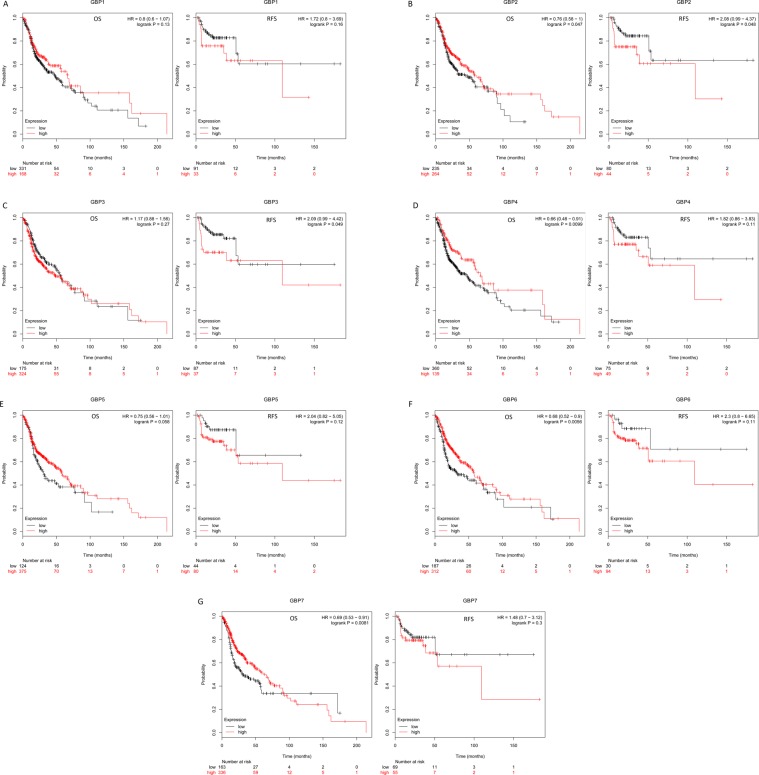
The Prognostic Value of mRNA Level of *GBP* Factors in HNSCC Patients. The patient samples are split into two groups according to various quantile expressions of the proposed biomarker and gene expression data and relapse free survival (RFS) and overall survival (OS) information are downloaded from GEO, EGA and TCGA. (Kaplan-Meier Plotter Database).

We next used Kaplan-Meier plotter to explored the prognostic survival values of the mRNA expression of *GBPs* in HNSCC patients by using publicly free online datasets. The Kaplan-Meier curve and log rank test analyses were shown in Fig. 5, higher mRNA expression of *GBP2* (HR = 2.08, 95% CI: 0.99–4.37, *P* = 0.048) and *GBP3* (HR = 2.09, 95% CI: 0.99–4.42, *P* = 0.049) were significantly associated with shorter relapse free survival (RFS) of HNSCC patients. Interestingly, higher mRNA expression of *GBP2* (HR = 0.76, 95% CI: 0.58–1, *P* = 0.047), *GBP4* (HR = 0.66, 95% CI: 0.48–0.91, *P* = 0.0099), *GBP6* (HR = 0.68, 95% CI: 0.52–0.9, *P* = 0.0056) and *GBP7* (HR = 0.69, 95% CI: 0.53–0.91, *P* = 0.0081) were significantly associated with longer overall survival (OS) of HNSCC patients. These results suggested that mRNA level of *GBP2/3/4/6/7* plays an important role in cancer patients’ prognosis and they may be exploited as novel useful biomarkers for prediction of HNSCC patients’ survival. We then tried to evaluate the independent prognostic value of expression of *GBPs* involve in OS in HNSCC patients from TCGA database. Significant high expression of *GBP1/3/4/5/6/7* in HNSCC tissues compared to normal tissues (Supplementary Fig.). In univariate analysis, we found that tumor stage (HR = 1.80, 95% CI: 1.23–2.66, *P* < 0.05), T-stage (HR = 1.36, 95% CI: 1.05–1.76, *P* < 0.05), N-stage (HR = 1.36, 95% CI: 1.10–1.68, *P* < 0.05), high expressions of *GBP1/6/7* were linked to shorter OS of HNSCC patients. Multivariate analysis suggested that N-stage (HR = 1.33, 95% CI: 1.03–1.72, *P* < 0.05), high expressions of *GBP1* and low expression of *GBP6/7* were linked to shorter OS of cancer patients (Supplementary Table 1). These results showed that transcriptional expressions of *GBP6/7* were independent prognostic factors for patients with HNSCC.

### Predicted Functions and Pathways of GBPs in HNSCC

**Figure 6:**
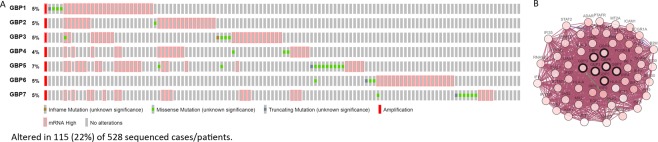
*GBPs* Gene Expression and Mutation Analysis in HNSCC (cBioPortal Database). (**A**) *GBPs* gene expression and mutation analysis; (**B**) The protein- protein interaction network for *GBPs* and the 50 most frequently altered neighbor genes.

We next explored the *GBP*s alterations and networks via the cBioPortal online tool and we further analyzed 50 neighbor genes which were significantly related to *GBPs* mutations. In the 528 sequenced HNSCC patients, genetic alteration was found in 115 patients and the mutation rate was 22%, *GBP5* ranked the highest genes with genetic alterations with the mutation rates were 7% and we also shown the network for *GBPs* and the 50 most frequently altered neighbor genes (Fig. 6). The top 10 type and frequency of *GBPs* neighbor gene alterations in HNSCC including *ADAR*, *IRF6, IRF9, IRF2, CD44, STAT1, FCGR1A, NFATC2, ICAM1* and *STAT2* (Table 2). In addition, functions and pathways of *GBPs* and their 50 frequently altered neighbor genes were analyzed by GO and KEGG in DAVID online database (Supplementary Table 3). GO analysis results showed that changes in biological processes (BP) were significantly enriched in cellular response to type I interferon, regulation of immune response, multi-organism process, response to virus and cytokine production *et al*. Changes in molecular function (MF) were mainly enriched in oligoadenylate synthetase activity, protein binding, regulatory region DNA binding and nucleic acid binding. Changes in cell component (CC) were mainly enriched in intracellular membrane-bounded organelle, cytoplasmic part, membrane-bounded organelle and MHC class I protein complex *et al*.

**Table 2:** The top 10 type and frequency of *GBPs* neighbor gene alterations in HNSCC (cBioPortal).

Gene Symbol	Amplification	Up-regulation	Down-regulation	Mutation	Total Alteration
*ADAR*	1.3	6.2	0.4	1.5	9.1
*IRF6*	0.9	6.6	0.4	0.9	8.7
*IRF9*	0.6	6.0	1.5	0.4	8.7
*IRF2*	0.4	2.1	2.8	1.5	8.4
*CD44*	3.4	6.6	0.4	0.6	8.5
*STAT1*	2.8	4.9	0	2.8	8.5
*FCGR1A*	2.1	4.2	0	0.2	7.9
*NFATC2*	0.2	4.3	0	2.8	7.5
*ICAM1*	0.9	4.0	0	1.1	7.0
*STAT2*	0.4	5.1	0	0.9	6.4

KEGG were mainly enriched in antigen processing and presentation and viral infection.

### Immune infiltrates in correlation with GBPs in HNSCC

Correlation between *GBPs* in HNSCC expression and abundance of immune infiltrates was statistically significant (*P* < 0.05, Supplementary Table. 2**)**. Cumulative survive showed that B cells of immune infiltrates statistically significant (*P* < 0.05) of *GBPs* in HNSCC and it is worth further research and exploration (Supplementary Table 4**)**.

## Discussion

Although certain *GBPs* members have been shown to play a key role in cancer, the different roles of *GBP* family members in HNSCC remain to be elucidated. So, we first explored the mutations and expressions of various *GBPs* based on thousands of variations in copy numbers or gene expressions in patients whom with HNSCC. Our study showed that high expressions of mRNA in all the *GBPs* family members, and mRNA expression of *GBPs* was significantly related to patients’ individual cancer stages in HNSCC patients. Multivariate analysis suggested that N-stage (HR = 1.33, 95% CI: 1.03–1.72, *P* < 0.05), high expressions of *GBP1* and low expression of *GBP6/7* were linked to shorter OS of cancer patients.

The occurrence of HPV-related HNSCC has steadily rised over the past two decades and now indicate a majority of HNSCC cases^23^. Epstein–Barr virus (EBV) infection is found in 90% to 100% of nasopharyngeal carcinoma (NPC) cases in endemic regions^24^. *GBPs* belongs to the IFNs induced guanylate-binding protein family, while IFNs are the main antiviral factors released during the process of infecting host cells by viruses or other pathogens, and have cell surface receptors that bind to responsive cells, regulating host antiviral status, anti-malignant cell proliferation and immune response^15^. Functions and pathways of GBPs and their 50 frequently altered neighbor genes were analyzed by GO and KEGG demonstrated that changes in BP of DEGs were significantly enriched in regulation of immune response and virus and cytokine production, MF were mainly enriched in protein/region DNA/nucleic acid binding and CC were mainly enriched in intracellular membrane-bounded organelle and MHC class I protein complex *et al*. KEGG were mainly enriched in antigen processing and presentation and viral infection. So, we presume that *GBPs* play a synergistic role in the HPV-HNSCC or other viral related cancers.

*GBP1* expression is excited not only by type I and type II interferons (IFN), including IFN-γ, and also by interleukin-1α, IL- 1β, and tumor necrosis factor-α^25–28^. *In vivo*, *GBP1* is related to the existence of inflammation and has been adhere to in inflammatory bowel diseases (IBD), autoimmune diseases and cancers. *GBP1* regulates the suppression of endothelial cells invasion and proliferation in reaction to inflammatory cytokines and exhibit antiviral activity^27^. Yu *et al*. reported that *GBP1* positively regulating the invasion and migration of oral cavity squamous cell carcinoma cells^29^. A recent study showed that *GBP1* may promote lung adenocarcinoma invasiveness by stimulate cell motility and via manage of *GBP1* expression may development of new therapeutic ways for tumor^30^. *GBP1* also found as a fetal T lymphocyte-induced protein that cause breast cancer cells to cross the blood-brain barrier^31^. Our results showed that higher mRNA expressions of *GBP1* were found in HNSCC tissues, and that mRNA expression of *GBP1* was significantly related with patients’ cancer stages and abundance of immune infiltrates. Moreover, previous studies focus on the role of T immune cell in cancers, while our cumulative survive showed that B cells significantly stimulant the prognosis and might have a medical prognostic significance linked to *GBPs* in HNSCC. *GBP2* is strongly induced by IFN-γ. *GBP2* is obviously higher in esophageal squamous cell carcinomas than in adjacent normal epithelium cells and is consistently increased in a p53-dependent manner and also followed by an rise in protein levels of IRF-1^32^. Zhang *et al*.^33^ first found that *GBP2* inhibits cell metastasis and mitochondrial fission in breast cancer cells not only *in vitro* but *in vivo* and another study found an interesting result that *GBP2* related to a recently generally T cell signature, suggesting tumor infiltration with T cells in breast cancer^34^. Other study found *GPB2/5* shown wide antiviral activity by suppressing the activation of the virus-dependency factor furin^35^. *GBP2* was similarly relocalized in cells in which autophagy was impaired^36^. Our study results shown that higher mRNA expression of *GBP2* were significantly related to longer OS and RFS of HNSCC patients. So, we guess *GBP2* may play a critical role in cancer autophagy and needs more study to convince.

Xu *et al*.^37^ first found that *GBP3* lead to the proliferation of glioma cells via *SQSTM1-ERK1/2* pathway. *GBP3* also has been reported to contributed to anti-influenza activity via suppressing viral transcription and replication^38^. *GBP1* and *GBP3* exhibit highest homology between the seven members (88% amino acid sequence)^12^ so *GBP3* may be a novel proto-oncogene. Similarly, our results showed that higher mRNA expressions of *GBP3* and related to shorter OS in HNSCC tissues. Study reported that *GBP4* is a negative regulator of virus-induced IFN-I production resulting in impaired *TRAF6*-mediated *IRF7* ubiquitination and it is deemed as a novel protein targeting *IRF7* and suppressing its function^39^. Therefore, *GBP4* may affect ubiquitination-mediated pathways, thereby inhibiting viral infection. *IRF* regulation of tumors is mainly through regulation of the cell cycle, apoptosis or tumor suppressor gene p53, but also regulation of anti-tumor immunity, or regulation of interferon signaling Pathway (eg *IRF9*)^40,41^. Our results found the 50 most frequently altered *GBPs* neighbor gene alterations in HNSCC including interferon regulatory factors 6 (*IRF6*), *IRF9* and *IRF2*, indicated that *IRFs* and *GBPs* coordinate the development of tumors and requires more study. *GBP5* have splice variants (*GBP5a, GBP5b, GBP5ta*) before 1992^42^. Krapp *et al*.^43^ reported that *GBP5* has a broad spectrum of antiretroviral capabilities. Wehner *et al*.^44^ reported that only the a/b splice variant is rise in healthy cells, but the truncated splice variant has been discovered in each melanoma and lots of lymphoma cell lines detected. Study also found *GBP5* act as an only rheostat for *NLRP3* inflammasome activation and thus alert the immune system to the exist of infection or tissue damage^45^. Our results showed that higher mRNA expressions of *GBP5* in HNSCC tissues and may related with patients’ tumor immune microenvironment. Until now, about the function of *GBP6/7* is rare. *HuGBP-6* lacks a GAS or NF-κB site and therefore, would not be expected to be induced by IFNs^46^. In GEPIA database analysis we found high *GBP6* expression in normal tissue and related with patients’ cancer stages, while in TCGA database analysis we found high expression in cancer tissue, in addition, in HPA databased we found no expression and low protein expression of *GBP6* was observed in normal and tumor tissues. Both *GBP6/7* significantly related to longer OS of HNSCC patients. *GBP7* can triggered an *IFIT5-IRF1/3-RSAD5* pathway in the DF-1 cells which potentially limited the viral replication cycle in the initial infection stage^47^. Thus, more researches should conduct to provide new ideas for the development of anti-tumor drugs and the treatment of human diseases.

There were some limitations in our study, one is that all the data analyzed in our study was retrieved from the online databases and cannot provides precise clinical data, larger sample sizes are needed to validate our findings, another is that we did not evaluate the potential diagnostic and therapeutic roles of *GBPs* and tumor markers can vary widely due to the histological type of HNSCC and multiple anatomical sites, so future related researches are required. Finally, due to insufficient data, we were unable to compare differences in function of *GBPs* between HPV-positive and HPV-negative HNSCC patients and we will do a lot of work in the future. In conclusion, our results showed that over expressions of 7 *GBPs* members and multivariate analysis suggested that N-stage, high expressions of *GBP1* and low expression of *GBP6/7* were linked to shorter OS in HNSCC patients. In addition, B-cell infiltration of immune cells affects prognosis and may be related to *GBPs* in HNSCC. We hypothesized that the *GBPs* plays a synergistic role in virus-associated HNSCC. This research uses online tools that rely on the most popular bioinformatics theories to perform target gene analysis of tumor data information from open databases, and authorizes large-scale HNSCC genomics research and follow-up functional exploration.

## Materials and Methods

### Ethics statement

As the work is a bioinformatics analysis article, so the ethical approval was not necessary and all the data were retrieved from the free online databases.

### ONCOMINE database

The DNA copy number and mRNA expression of *GBPs* in HNSCC were investigated inside the Oncomine 4.5 database. Oncomine (www.oncomine.org) contains 715 gene expression data sets and 86,733 samples, is also the biggest oncogene chip database and incorporated data mining platform worldwide^48^. This analysis drew on a series of HNSCC studies and *GBPs* expression was involved in evaluated in HNSCC tissue in respect to its expression in normal tissue, and *P* < 0.05 as the cutoff criterion considered statistically significant.

### Human protein atlas

The Human Protein Atlas (https://www.proteinatlas.org/about/licence) is a website that involve immunohistochemistry-based expression data for distribution and expression of 20 tumor tissues, 47 cell lines, 48 human normal tissues and 12 blood cells, the results are represented by at least 576 immunohistochemical staining maps, which have been read and indexed by professionals. These tissues were from 144 different individuals and 216 tumor tissues, which ensured that the staining results were fully representative^49^. In our study, direct contrast of protein expression of different *GBPs* family members between normal and HNSCC tissues was used by immunohistochemistry image.

### GEPIA dataset

GEPIA^50^ is a newly created online interactive web server which enables users to exploring the RNA sequencing expression information of tumors/normal tissues or samples from the Genotype Tissue Expression (GTEx) projects and The Cancer Genome Atlas (TCGA), based on a criterion processing pipeline. GEPIA offer customizable functions such as profiling regarding to pathological stages, cancer types, differential expression analysis, survival analysis, correlation analysis and similar gene detection.

### Kaplan-meier plotter

The prognostic significance of mRNA expression of different *GBPs* in HNSCC was evaluated by using Kaplan-Meier plotter (http://kmplot.com/analysis/)^51^, in which data about gene expression with survival of patients in 21 cancer types. The system includes gene chip and RNA-seq information - sources for the databases include TCGA, GEO and EGA. By parameter setting, we can analyze the prognosis of patients in different subgroups, different pathological parameters, different treatment modes, and different data sets. In Kaplan-Meier plotter, cancer patient samples were split into low and high expression group according to median values of mRNA expression and assessed by K-M survival plot.

### TCGA database

TCGA uses large-scale genome sequencing to map out the genomic variation maps of all human cancers, and conduct systematic analysis to understand the mechanism of cancer cell occurrence and development, and on this basis, to obtain new diagnostic and therapeutic methods^52^. In our analysis, clinicopathological parameters of 512 HNSCC patients and mRNA expression of *GBPs* of 529 HNSCC patients were downloaded from the TCGA. The listwise deletion technique was utilized to deal with missing data, which excluded the entire sample from the investigation if any single value was absent. Cox analysis was utilized to evaluate the impact of *GBPs* expression on survival alongside other clinical attributes (such as age, gender, stage, distant metastasis).

### cBioPortal

The cBioPortal (http://cbioportal.org)^53^ is an open-access asset gives visualization, analysis and download of substantial scale cancer genomics data sets. We utilized c-BioPortal to analyze *GBPs* alterations in the TCGA HNSCC samples and shows an overview of genetic alterations per test in *GBPs*. A tab biological interaction network of the *GBPs* and their co-expression genes was analyzed and neighboring genes with alteration frequencies were included. Functions of *GBPs* mutations and 50 genes significantly related to *GBPs* mutations were performed by GO and KEGG in the Database for Annotation, Visualization, and Integrated Discovery (DAVID) online tool. *P*-Value<0.05 as statistically significant.

### TIMER analysis

TIMER (https://cistrome.shinyapps.io/timer/)^54^ is a comprehensive asset for systematical investigation of immune infiltrates over various malignancy types. The abundances of six immune infiltrates (CD8+ T cells, B cells, CD4+ T cells, Macrophages, Neutrphils and Dendritic cells) are assessed by our statistical method, which is approved using pathological estimations. Using Gene module to explore correlation between *GBPs* expression and abundance of immune infiltrates in HNSCC; Survival module to draw Kaplan-Meier plots for immune infiltrates and *GBPs* to picture the survival differences. *P*-Value < 0.05 as statistically significant.

## Supplementary information

Supplement.
