# Supplementary material for: Comprehensive Analysis of the Expression and Prognosis for GBPs in Head and neck squamous cell carcinoma
Source: Sci Rep. 2020 Apr 8;10:6085. doi: 10.1038/s41598-020-63246-7 (PMC7142114; doi:10.1038/s41598-020-63246-7)
Supplement: Supplementary file 1 — Supplement. [file 41598_2020_63246_MOESM1_ESM.docx]

**Comprehensive Analysis of the Expression and Prognosis for GBPs in Head and neck squamous cell carcinoma**

**Zeng-Hong Wu, MB; Fu-Cheng Cai, MB; Yi Zhong, PhD.**


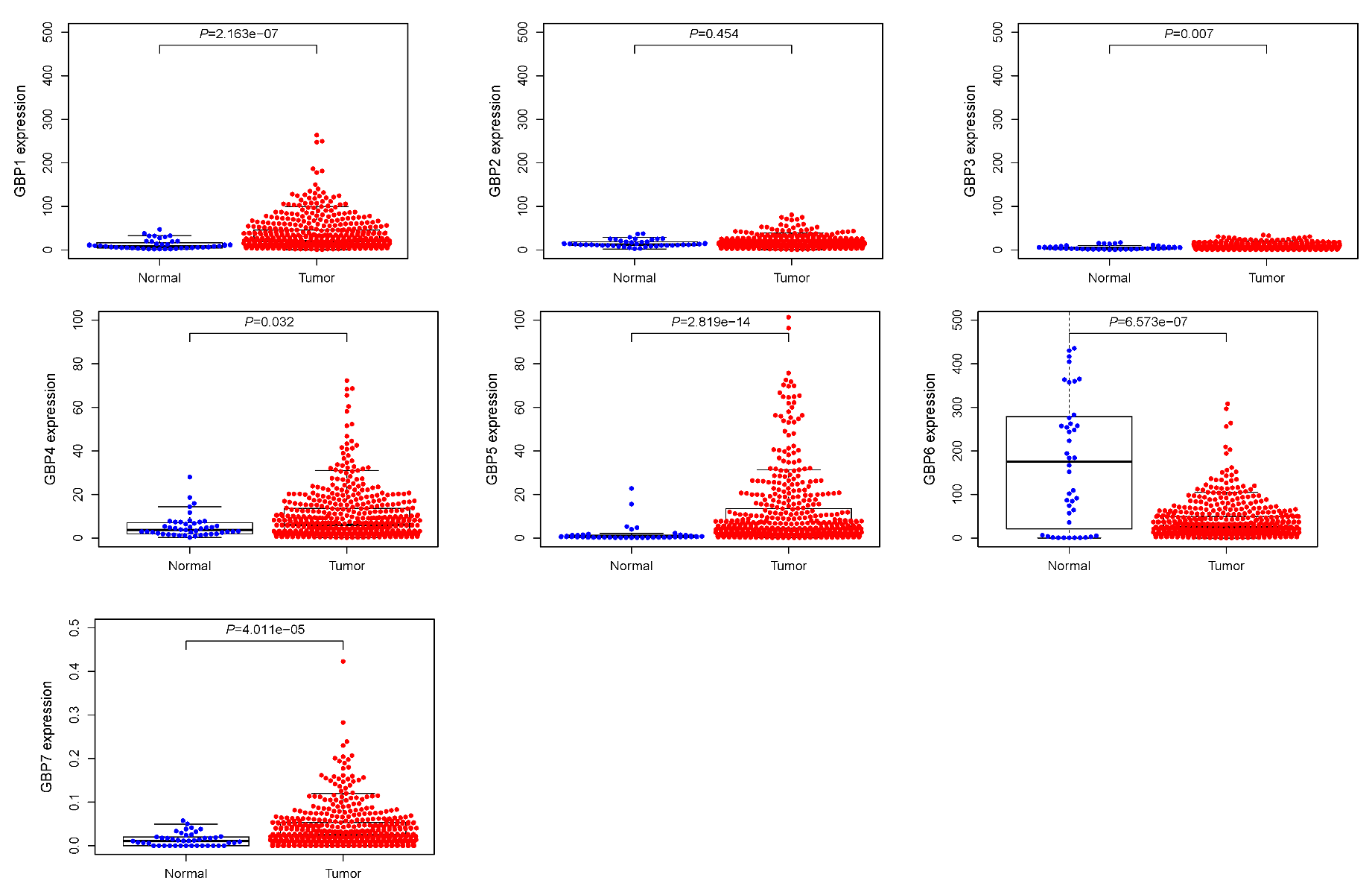


Supplementary Figure. The expression of *GBPs* involves in OS in HNSCC patients from TCGA database. Significant high expression of *GBP1/3/4/5/6/7* in HNSCC tissues compared to normal tissues. The patient samples are split into two groups according to various quantile expressions of the proposed biomarker and *P*-Value<0.05 as statistically significant. (TCGA Database).

Supplementary Table 1. a. Associations with overall survival and clinicopathologic characteristics in TCGA patients using Cox regression. b. Multivariate survival model after variable selection.

| Clinicopathologic variable | HR (95% CI) | p-Value |
| --- | --- | --- |
| a. |  |  |
| Age (continuous) | 1.01 (0.98-1.03) | 0.538 |
| Gender (Female vs. Male) | 1.14 (0.28-4.70) | 0.852 |
| Grade (continuous) | 1.32 (0.88-2.00) | 0.186 |
| Stage (continuous) | 1.80 (1.23-2.66) | 0.003 |
| T-stage (continuous) | 1.36 (1.05-1.76) | 0.021 |
| Distant metastasis (positive vs. negative) | 0.96 (0.69-1.35) | 0.823 |
| Lymph nodes (positive vs. negative) | 1.36 (1.10-1.68) | 0.004 |
| GBP1 expression (low vs. high) | 1.00 (0.99-1.01) | 0.041 |
| GBP2 expression (low vs. high) | 1.00 (0.98-1.03) | 0.980 |
| GBP3 expression (low vs. high) | 1.03 (0.98-1.07) | 0.225 |
| GBP4 expression (low vs. high) | 0.99 (0.96-1.02) | 0.369 |
| GBP5 expression (low vs. high) | 1.00 (0.98-1.02) | 0.940 |
| GBP6 expression (low vs. high) | 1.00 (0.99-1.00) | 0.034 |
| GBP7 expression (low vs. high) | 0.000047 (0.00000023.-0.09) | 0.010 |
| b. |  |  |
| Lymph nodes (positive vs. negative) | 1.33 (1.03-1.72) | 0.027 |
| GBP1 expression (low vs. high) | 1.00 (0.99-1.00) | 0.048 |
| GBP2 expression (low vs. high) | 1.00 (0.97-1.03) | 0.952 |
| GBP3 expression (low vs. high) | 1.03 (0.99-1.07) | 0.160 |
| GBP4 expression (low vs. high) | 0.99 (0.95-1.02) | 0.388 |
| GBP5 expression (low vs. high) | 1.00 (0.99-1.02) | 0.710 |
| GBP6 expression (low vs. high) | 0.99 (0.99-1.00) | 0.041 |
| GBP7 expression (low vs. high) | 0.0001 (0.00000044.-0.40) | 0.029 |
|  |  |  |

| variable | partial.cor | p |
| --- | --- | --- |
| GJB1 |  |  |
| Purity | -0.34214901 | 5.51E-15 |
| B Cell | -0.022573443 | 0.623241082 |
| CD8+ T Cell | 0.317410397 | 1.48E-12 |
| CD4+ T Cell | 0.359105744 | 4.68E-16 |
| Macrophage | 0.210136911 | 3.19E-06 |
| Neutrophil | 0.707893332 | 4.84E-74 |
| Dendritic Cell | 0.459524847 | 1.49E-26 |
| GJB2 |  |  |
| variable | partial.cor | p |
| Purity | -0.294218448 | 2.66124E-11 |
| B Cell | 0.121903811 | 0.007754617 |
| CD8+ T Cell | 0.467895144 | 3.68768E-27 |
| CD4+ T Cell | 0.376291329 | 1.36012E-17 |
| Macrophage | 0.345266066 | 5.72856E-15 |
| Neutrophil | 0.585078094 | 2.45796E-45 |
| Dendritic Cell | 0.457885021 | 2.35791E-26 |
| GJB3 |  |  |
| variable | partial.cor | p |
| Purity | -0.199935428 | 7.70E-06 |
| B Cell | -0.001683845 | 0.970771569 |
| CD8+ T Cell | 0.046802729 | 0.309232203 |
| CD4+ T Cell | 0.245142421 | 5.33E-08 |
| Macrophage | 0.134586697 | 0.003039866 |
| Neutrophil | 0.40194333 | 5.03E-20 |
| Dendritic Cell | 0.269832892 | 1.74E-09 |
| GJB4 |  |  |
| variable | partial.cor | p |
| Purity | -0.252062976 | 1.39195E-08 |
| B Cell | 0.211870615 | 3.11084E-06 |
| CD8+ T Cell | 0.556697252 | 6.28904E-40 |
| CD4+ T Cell | 0.556472775 | 2.24044E-40 |
| Macrophage | 0.396034378 | 1.3777E-19 |
| Neutrophil | 0.797063362 | 1.5111E-106 |
| Dendritic Cell | 0.653139484 | 5.97492E-60 |
| GJB5 |  |  |
| variable | partial.cor | p |
| Purity | -0.356165777 | 3.43E-16 |
| B Cell | 0.037805669 | 0.410537621 |
| CD8+ T Cell | 0.424731138 | 3.49E-22 |
| CD4+ T Cell | 0.366730989 | 1.00E-16 |
| Macrophage | 0.290606376 | 7.45E-11 |
| Neutrophil | 0.696172791 | 1.13E-70 |
| Dendritic Cell | 0.504413254 | 1.79E-32 |
| GJB6 |  |  |
| variable | partial.cor | p |
| Purity | -0.110554134 | 0.014049252 |
| B Cell | 0.024716812 | 0.590631224 |
| CD8+ T Cell | 0.065940736 | 0.151743181 |
| CD4+ T Cell | 0.082837593 | 0.069790762 |
| Macrophage | -0.100857668 | 0.026658206 |
| Neutrophil | 0.037167413 | 0.417019081 |
| Dendritic Cell | -0.028763832 | 0.52870361 |
| GJB7 |  |  |
| variable | partial.cor | p |
| Purity | -0.108066499 | 0.0163768 |
| B Cell | 0.180257304 | 7.66E-05 |
| CD8+ T Cell | 0.336647727 | 5.04E-14 |
| CD4+ T Cell | 0.370580868 | 4.51E-17 |
| Macrophage | 0.285866438 | 1.55E-10 |
| Neutrophil | 0.506314312 | 1.50E-32 |
| Dendritic Cell | 0.453664997 | 7.63E-26 |
| variable | partial.cor | p |
|  |  |  |

Supplementary Table 2. Correlation between *GBPs* in HNSCC expression and abundance of immune infiltrates was statistically significant. The scatterplots showing the purity-corrected partial Spearman’s correlation and statistical significance. (TIMER Database)

| Supplementary Table 3. Functions and pathways of GBPs and their 50 frequently altered neighbor genes were analyzed by GO and KEGG in DAVID online database. | | | | | | |
| --- | --- | --- | --- | --- | --- | --- |
| Category | Term | Count | % | PValue | Fold Enrichment | FDR |
| KEGG |  |  |  |  |  |  |
| KEGG_PATHWAY | hsa05164:Influenza A | 13 | 0.238357 | 6.83E-12 | 15.57419 | 6.89E-09 |
| KEGG_PATHWAY | hsa05168:Herpes simplex infection | 13 | 0.238357 | 1.24E-11 | 14.80825 | 1.26E-08 |
| KEGG_PATHWAY | hsa05162:Measles | 10 | 0.183352 | 5.54E-09 | 15.67327 | 5.59E-06 |
| KEGG_PATHWAY | hsa05160:Hepatitis C | 10 | 0.183352 | 5.54E-09 | 15.67327 | 5.59E-06 |
| KEGG_PATHWAY | hsa04612:Antigen processing and presentation | 6 | 0.110011 | 2.30E-05 | 16.45694 | 0.0232 |
| BP |  |  |  |  |  |  |
| GOTERM_BP_ALL | GO:0071357~cellular response to type I interferon | 37 | 0.678401 | 3.51E-79 | 171.3469 | 5.98E-76 |
| GOTERM_BP_ALL | GO:0060337~type I interferon signaling pathway | 37 | 0.678401 | 3.51E-79 | 171.3469 | 5.98E-76 |
| GOTERM_BP_ALL | GO:0034340~response to type I interferon | 37 | 0.678401 | 4.46E-78 | 162.5599 | 7.60E-75 |
| GOTERM_BP_ALL | GO:0019221~cytokine-mediated signaling pathway | 48 | 0.880088 | 1.54E-69 | 29.69189 | 2.63E-66 |
| GOTERM_BP_ALL | GO:0071345~cellular response to cytokine stimulus | 48 | 0.880088 | 2.12E-64 | 23.2993 | 3.61E-61 |
| GOTERM_BP_ALL | GO:0034097~response to cytokine | 48 | 0.880088 | 1.02E-61 | 20.51036 | 1.74E-58 |
| GOTERM_BP_ALL | GO:0045087~innate immune response | 48 | 0.880088 | 7.58E-61 | 19.6762 | 1.29E-57 |
| GOTERM_BP_ALL | GO:0060333~interferon-gamma-mediated signaling pathway | 29 | 0.53172 | 7.11E-55 | 124.2265 | 1.21E-51 |
| GOTERM_BP_ALL | GO:0034341~response to interferon-gamma | 32 | 0.586725 | 4.72E-52 | 68.11307 | 8.05E-49 |
| GOTERM_BP_ALL | GO:0006955~immune response | 49 | 0.898423 | 1.13E-50 | 10.82656 | 1.93E-47 |
| GOTERM_BP_ALL | GO:0006952~defense response | 48 | 0.880088 | 1.98E-48 | 10.83617 | 3.38E-45 |
| GOTERM_BP_ALL | GO:0071346~cellular response to interferon-gamma | 29 | 0.53172 | 1.64E-47 | 73.61572 | 2.81E-44 |
| GOTERM_BP_ALL | GO:0071310~cellular response to organic substance | 48 | 0.880088 | 6.57E-41 | 7.524843 | 1.12E-37 |
| GOTERM_BP_ALL | GO:0002376~immune system process | 49 | 0.898423 | 6.78E-41 | 6.81217 | 1.16E-37 |
| GOTERM_BP_ALL | GO:0007166~cell surface receptor signaling pathway | 49 | 0.898423 | 3.19E-39 | 6.291495 | 5.45E-36 |
| GOTERM_BP_ALL | GO:0070887~cellular response to chemical stimulus | 48 | 0.880088 | 5.69E-37 | 6.211974 | 9.70E-34 |
| GOTERM_BP_ALL | GO:0010033~response to organic substance | 48 | 0.880088 | 1.15E-35 | 5.828953 | 1.96E-32 |
| GOTERM_BP_ALL | GO:0006950~response to stress | 49 | 0.898423 | 1.71E-32 | 4.566766 | 2.92E-29 |
| GOTERM_BP_ALL | GO:0051607~defense response to virus | 24 | 0.440044 | 9.24E-31 | 35.91552 | 1.58E-27 |
| GOTERM_BP_ALL | GO:0042221~response to chemical | 49 | 0.898423 | 4.02E-30 | 4.078698 | 6.87E-27 |
| GOTERM_BP_ALL | GO:0009615~response to virus | 24 | 0.440044 | 1.27E-27 | 26.44583 | 2.16E-24 |
| GOTERM_BP_ALL | GO:0044419~interspecies interaction between organisms | 32 | 0.586725 | 9.80E-27 | 10.98818 | 1.67E-23 |
| GOTERM_BP_ALL | GO:0044403~symbiosis, encompassing mutualism through parasitism | 32 | 0.586725 | 9.80E-27 | 10.98818 | 1.67E-23 |
| GOTERM_BP_ALL | GO:0043207~response to external biotic stimulus | 30 | 0.550055 | 3.66E-26 | 12.40147 | 6.25E-23 |
| GOTERM_BP_ALL | GO:0051707~response to other organism | 30 | 0.550055 | 3.66E-26 | 12.40147 | 6.25E-23 |
| GOTERM_BP_ALL | GO:0098542~defense response to other organism | 26 | 0.476714 | 4.60E-26 | 18.03652 | 7.84E-23 |
| GOTERM_BP_ALL | GO:0002252~immune effector process | 29 | 0.53172 | 5.01E-26 | 13.3937 | 8.54E-23 |
| GOTERM_BP_ALL | GO:0016032~viral process | 31 | 0.56839 | 1.06E-25 | 10.99742 | 1.80E-22 |
| GOTERM_BP_ALL | GO:0044764~multi-organism cellular process | 31 | 0.56839 | 1.31E-25 | 10.9183 | 2.23E-22 |
| GOTERM_BP_ALL | GO:0009607~response to biotic stimulus | 30 | 0.550055 | 1.60E-25 | 11.77642 | 2.73E-22 |
| GOTERM_BP_ALL | GO:0007165~signal transduction | 49 | 0.898423 | 4.94E-23 | 2.907706 | 8.43E-20 |
| GOTERM_BP_ALL | GO:0044700~single organism signaling | 49 | 0.898423 | 1.60E-21 | 2.705333 | 2.73E-18 |
| GOTERM_BP_ALL | GO:0023052~signaling | 49 | 0.898423 | 2.41E-21 | 2.682428 | 4.11E-18 |
| GOTERM_BP_ALL | GO:0007154~cell communication | 49 | 0.898423 | 2.70E-21 | 2.676016 | 4.61E-18 |
| GOTERM_BP_ALL | GO:0051716~cellular response to stimulus | 49 | 0.898423 | 3.16E-19 | 2.424137 | 5.39E-16 |
| GOTERM_BP_ALL | GO:0051704~multi-organism process | 35 | 0.641731 | 3.97E-19 | 5.123574 | 6.77E-16 |
| GOTERM_BP_ALL | GO:0048525~negative regulation of viral process | 13 | 0.238357 | 1.51E-16 | 41.25019 | 1.89E-13 |
| GOTERM_BP_ALL | GO:0045071~negative regulation of viral genome replication | 11 | 0.201687 | 1.55E-16 | 73.91437 | 1.89E-13 |
| GOTERM_BP_ALL | GO:0009605~response to external stimulus | 31 | 0.56839 | 3.25E-16 | 5.124703 | 5.66E-13 |
| GOTERM_BP_ALL | GO:0043901~negative regulation of multi-organism process | 14 | 0.256692 | 3.59E-16 | 30.55869 | 5.66E-13 |
| GOTERM_BP_ALL | GO:1903901~negative regulation of viral life cycle | 12 | 0.220022 | 9.29E-16 | 46.20592 | 1.51E-12 |
| GOTERM_BP_ALL | GO:0043903~regulation of symbiosis, encompassing mutualism through parasitism | 16 | 0.293363 | 1.24E-15 | 19.23895 | 2.09E-12 |
| GOTERM_BP_ALL | GO:0050896~response to stimulus | 49 | 0.898423 | 2.26E-15 | 2.016088 | 3.79E-12 |
| GOTERM_BP_ALL | GO:0043900~regulation of multi-organism process | 17 | 0.311698 | 3.53E-15 | 15.45304 | 6.06E-12 |
| GOTERM_BP_ALL | GO:0050792~regulation of viral process | 15 | 0.275028 | 1.07E-14 | 19.61988 | 1.84E-11 |
| GOTERM_BP_ALL | GO:0045069~regulation of viral genome replication | 11 | 0.201687 | 2.17E-14 | 46.53867 | 3.69E-11 |
| GOTERM_BP_ALL | GO:0035455~response to interferon-alpha | 8 | 0.146681 | 4.85E-14 | 144.2922 | 8.28E-11 |
| GOTERM_BP_ALL | GO:0019079~viral genome replication | 11 | 0.201687 | 3.49E-13 | 35.56257 | 5.95E-10 |
| GOTERM_BP_ALL | GO:1903900~regulation of viral life cycle | 12 | 0.220022 | 2.10E-12 | 23.23348 | 3.59E-09 |
| GOTERM_BP_ALL | GO:0050776~regulation of immune response | 19 | 0.348368 | 2.23E-11 | 7.139456 | 3.81E-08 |
| GOTERM_BP_ALL | GO:0048519~negative regulation of biological process | 36 | 0.660066 | 7.84E-11 | 2.668609 | 1.34E-07 |
| GOTERM_BP_ALL | GO:0050794~regulation of cellular process | 49 | 0.898423 | 1.49E-10 | 1.600915 | 2.54E-07 |
| GOTERM_BP_ALL | GO:0019058~viral life cycle | 14 | 0.256692 | 2.91E-10 | 10.54443 | 4.97E-07 |
| GOTERM_BP_ALL | GO:0042590~antigen processing and presentation of exogenous peptide antigen via MHC class I | 8 | 0.146681 | 6.77E-10 | 41.53865 | 1.16E-06 |
| GOTERM_BP_ALL | GO:0050789~regulation of biological process | 49 | 0.898423 | 1.04E-09 | 1.537447 | 1.78E-06 |
| GOTERM_BP_ALL | GO:0002682~regulation of immune system process | 20 | 0.366703 | 2.37E-09 | 4.973786 | 4.05E-06 |
| GOTERM_BP_ALL | GO:0035456~response to interferon-beta | 6 | 0.110011 | 6.29E-09 | 85.67347 | 1.07E-05 |
| GOTERM_BP_ALL | GO:0002474~antigen processing and presentation of peptide antigen via MHC class I | 8 | 0.146681 | 7.21E-09 | 29.79947 | 1.23E-05 |
| GOTERM_BP_ALL | GO:0048523~negative regulation of cellular process | 32 | 0.586725 | 1.47E-08 | 2.55028 | 2.50E-05 |
| GOTERM_BP_ALL | GO:0065007~biological regulation | 49 | 0.898423 | 1.52E-08 | 1.454105 | 2.59E-05 |
| GOTERM_BP_ALL | GO:0002479~antigen processing and presentation of exogenous peptide antigen via MHC class I, TAP-dependent | 7 | 0.128346 | 2.37E-08 | 38.0771 | 4.05E-05 |
| GOTERM_BP_ALL | GO:0001816~cytokine production | 13 | 0.238357 | 1.44E-07 | 7.060254 | 2.45E-04 |
| GOTERM_BP_ALL | GO:0019882~antigen processing and presentation | 9 | 0.165017 | 2.34E-07 | 13.58698 | 3.98E-04 |
| GOTERM_BP_ALL | GO:0044763~single-organism cellular process | 49 | 0.898423 | 2.66E-07 | 1.370104 | 4.54E-04 |
| GOTERM_BP_ALL | GO:0002478~antigen processing and presentation of exogenous peptide antigen | 8 | 0.146681 | 3.61E-07 | 16.92315 | 6.15E-04 |
| GOTERM_BP_ALL | GO:0019884~antigen processing and presentation of exogenous antigen | 8 | 0.146681 | 4.80E-07 | 16.2222 | 8.20E-04 |
| GOTERM_BP_ALL | GO:0001817~regulation of cytokine production | 12 | 0.220022 | 5.10E-07 | 7.127082 | 8.70E-04 |
| GOTERM_BP_ALL | GO:0045088~regulation of innate immune response | 10 | 0.183352 | 6.94E-07 | 9.519274 | 0.001184 |
| GOTERM_BP_ALL | GO:0048002~antigen processing and presentation of peptide antigen | 8 | 0.146681 | 7.08E-07 | 15.31593 | 0.001208 |
| GOTERM_BP_ALL | GO:0001819~positive regulation of cytokine production | 10 | 0.183352 | 1.24E-06 | 8.87808 | 0.002114 |
| GOTERM_BP_ALL | GO:0002480~antigen processing and presentation of exogenous peptide antigen via MHC class I, TAP-independent | 4 | 0.073341 | 1.82E-06 | 152.3084 | 0.003103 |
| GOTERM_BP_ALL | GO:0002684~positive regulation of immune system process | 14 | 0.256692 | 1.91E-06 | 4.997619 | 0.003254 |
| GOTERM_BP_ALL | GO:0060338~regulation of type I interferon-mediated signaling pathway | 5 | 0.091676 | 4.49E-06 | 43.93511 | 0.00766 |
| GOTERM_BP_ALL | GO:0019885~antigen processing and presentation of endogenous peptide antigen via MHC class I | 4 | 0.073341 | 7.80E-06 | 97.91254 | 0.013313 |
| GOTERM_BP_ALL | GO:0002483~antigen processing and presentation of endogenous peptide antigen | 4 | 0.073341 | 9.74E-06 | 91.38503 | 0.016607 |
| GOTERM_BP_ALL | GO:0042089~cytokine biosynthetic process | 6 | 0.110011 | 1.09E-05 | 19.96275 | 0.018652 |
| GOTERM_BP_ALL | GO:0042107~cytokine metabolic process | 6 | 0.110011 | 1.20E-05 | 19.58251 | 0.020486 |
| GOTERM_BP_ALL | GO:0019883~antigen processing and presentation of endogenous antigen | 4 | 0.073341 | 1.45E-05 | 80.63385 | 0.024719 |
| GOTERM_BP_ALL | GO:0031347~regulation of defense response | 11 | 0.201687 | 1.71E-05 | 5.57638 | 0.029164 |
| GOTERM_BP_ALL | GO:0044699~single-organism process | 49 | 0.898423 | 1.79E-05 | 1.255289 | 0.030529 |
| GOTERM_BP_ALL | GO:0050778~positive regulation of immune response | 11 | 0.201687 | 1.89E-05 | 5.511159 | 0.032281 |
| GOTERM_BP_ALL | GO:0009617~response to bacterium | 10 | 0.183352 | 2.00E-05 | 6.29952 | 0.034071 |
| GOTERM_BP_ALL | GO:0002486~antigen processing and presentation of endogenous peptide antigen via MHC class I via ER pathway, TAP-independent | 3 | 0.055006 | 2.40E-05 | 342.6939 | 0.040864 |
| CC |  |  |  |  |  |  |
| GOTERM_CC_ALL | GO:0043231~intracellular membrane-bounded organelle | 46 | 0.843418 | 1.79E-07 | 1.550362 | 2.23E-04 |
| GOTERM_CC_ALL | GO:0005829~cytosol | 26 | 0.476714 | 1.88E-07 | 2.844918 | 2.33E-04 |
| GOTERM_CC_ALL | GO:0044444~cytoplasmic part | 39 | 0.715072 | 8.86E-07 | 1.792932 | 0.001101 |
| GOTERM_CC_ALL | GO:0043227~membrane-bounded organelle | 47 | 0.861753 | 1.30E-06 | 1.431744 | 0.001616 |
| GOTERM_CC_ALL | GO:0042612~MHC class I protein complex | 4 | 0.073341 | 2.79E-06 | 135.243 | 0.003462 |
| GOTERM_CC_ALL | GO:0043229~intracellular organelle | 46 | 0.843418 | 7.24E-06 | 1.414021 | 0.008995 |
| GOTERM_CC_ALL | GO:0031901~early endosome membrane | 6 | 0.110011 | 1.43E-05 | 18.9111 | 0.01779 |
| GOTERM_CC_ALL | GO:0044428~nuclear part | 25 | 0.458379 | 1.60E-05 | 2.335584 | 0.019816 |
| GOTERM_CC_ALL | GO:0005737~cytoplasm | 43 | 0.788412 | 1.80E-05 | 1.484222 | 0.022407 |
| GOTERM_CC_ALL | GO:0043226~organelle | 47 | 0.861753 | 2.94E-05 | 1.328886 | 0.036502 |
| GOTERM_CC_ALL | GO:0030662~coated vesicle membrane | 6 | 0.110011 | 3.27E-05 | 15.93936 | 0.040598 |
| MF |  |  |  |  |  |  |
| GOTERM_MF_ALL | GO:0001730~2'-5'-oligoadenylate synthetase activity | 4 | 0.073341 | 8.08E-08 | 351.6875 | 1.03E-04 |
| GOTERM_MF_ALL | GO:0005515~protein binding | 45 | 0.825083 | 2.49E-06 | 1.464551 | 0.003178 |
| GOTERM_MF_ALL | GO:0000975~regulatory region DNA binding | 13 | 0.238357 | 2.70E-06 | 5.341048 | 0.00345 |
| GOTERM_MF_ALL | GO:0001067~regulatory region nucleic acid binding | 13 | 0.238357 | 2.73E-06 | 5.334816 | 0.003493 |

Supplementary Table 4. Cumulative survive showed that B cells of immune infiltrates statistically significant (*P*<0.05) of *GBPs* in HNSCC indicating that B cells significantly affecting the prognosis (TIMER Database).

| Cancer | Variable | *P*-value |
| --- | --- | --- |
| HNSCC | B Cell | 0.044719315 |
| HNSCC | CD8+ T Cell | 0.362878638 |
| HNSCC | CD4+ T Cell | 0.137665941 |
| HNSCC | Macrophage | 0.616092495 |
| HNSCC | Neutrophil | 0.061422121 |
| HNSCC | Dendritic Cell | 0.296953952 |
| HNSCC | *GBP1* | 0.980603449 |
| HNSCC | *GBP2* | 0.414009127 |
| HNSCC | *GBP3* | 0.911896766 |
| HNSCC | *GBP4* | 0.066513818 |
| HNSCC | *GBP5* | 0.115771686 |
| HNSCC | *GBP6* | 0.389510707 |
| HNSCC | *GBP7* | 0.126761463 |
|  |  |  |
